# Recurrence of SARS-CoV-2 nucleic acid positive test in patients with COVID-19: a report of two cases

**DOI:** 10.1186/s12890-020-01348-8

**Published:** 2020-11-23

**Authors:** Jian Wu, Juan Cheng, Xiaowei Shi, Jun Liu, Biao Huang, Xinguo Zhao, Yuanwang Qiu, Jiong Yu, Hongcui Cao, Lanjuan Li

**Affiliations:** 1 State Key Laboratory for the Diagnosis and Treatment of Infectious Diseases, National Clinical Research Center for Infectious Diseases, The First Affiliated Hospital, Zhejiang University School of Medicine, 79 Qingchun Rd, Hangzhou, 310003 China; 2 Department of Laboratory Medicine, Yancheng Clinical Medical College of Nanjing Medical University, Yancheng, 224001 China; 3 Department of Infectious Disease, The Second People’s Hospital of Yancheng City, Yancheng, 224005 China; 4 Department of Laboratory Medicine, The Fifth People’s Hospital of Wuxi, Wuxi, 214005 China; 5 College of Life Sciences and Medicine, Zhejiang Sci-Tech University, Hangzhou, 310018 China; 6 Department of Respiration, The Fifth People’s Hospital of Wuxi, Wuxi, 214005 China; 7 Department of Infectious Diseases, The Fifth People’s Hospital of Wuxi, Wuxi, 214005 China; 8 Zhejiang Provincial Key Laboratory for Diagnosis and Treatment of Aging and Physic-chemical Injury Diseases, 79 Qingchun Rd, Hangzhou, 310003 China

**Keywords:** Severe acute respiratory syndrome coronavirus 2 (SARS-CoV-2), Coronavirus disease-19 (COVID-19), Recurrence, Nucleic acid test

## Abstract

**Background:**

The recurrence of positive SARS-CoV-2 nucleic acid test results in patients with COVID-19 is becoming more important and warrants more attention.

**Case presentation:**

This study reports 2 cases, a child with mild COVID-19 and an adult female with moderate COVID-19, who were discharged after three consecutive negative nucleic acid tests and were later readmitted to the hospital for recurrence of SARS-CoV-2 nucleic acid positivity. By tracking the patients’ symptoms, serum antibodies, and imaging manifestations after readmission, we found that they showed a trend of gradual improvement and recovery throughout treatment. They were cured without additional treatment, with the appearance of antibodies and the recovery of immune functions.

**Conclusions:**

It is deemed extremely necessary to improve the discharge standard of care. At the same time, nucleic acid detection is recommended to increase the dynamic monitoring of serum antibodies and imaging, strengthen the management of discharged patients, and appropriately extend the home or centralized isolation time.

**Supplementary Information:**

The online version contains supplementary material available at 10.1186/s12890-020-01348-8.

## Background

To date, there are a few reports about the recurrence of severe acute respiratory syndrome coronavirus 2 (SARS-CoV-2) nucleic acid positivity in patients with coronavirus disease 2019 (COVID-19) after discharge [1–3]. Although patients should be isolated for 14 days after discharge according to the guidelines from the World Health Organization (WHO) and National Health Commission of the People’s Republic of China [4, 5], the existing discharge standard of care and the cause of recurrence of viral nucleic acid positivity have received increasing attention [6]. The standard procedures for management of contacts and cases of COVID-19 are as follows: novel coronavirus nucleic acid detection should be carried out within 2 h, and the new type of coronavirus nucleic acid test should be collected to ensure that suspected patients are transferred to the designated hospital as soon as possible. Novel coronavirus aetiological tests are recommended for patients having close contact with those infected with the new coronavirus. Suspected cases can be excluded only if the nucleic acid test for respiratory tract pathogens is negative on two consecutive occasions (sampling interval is at least 1 day). The discharge standard of care was as follows: the body temperature returned to normal for more than 3 days, respiratory symptoms improved significantly, and the nucleic acid test of respiratory tract pathogens was negative on two consecutive occasions (the sampling interval was at least 1 day). The isolated patient could be released from the hospital or transferred to the corresponding department for the treatment of other diseases according to the condition.

Recently, it has been reported that the faeces of some discharged patients can test positive with the nucleic acid test, without live virus being found in faecal culture [7]. Therefore, further studies are required to determine whether patients with nucleic acid positivity recurrence are infectious, they need to be readmitted to the hospital for treatment, and their families need to be isolated again. We conducted a retrospective study of two COVID-19 patients who showed recurrence of SARS-CoV-2 nucleic acid positivity in China. The two cases, an 8-year-old male and a 46-year-old female, were both imported cases. The epidemiology of the patients is shown in Figure S1. The study was approved by the ethics committee of the Fifth People’s Hospital of Wuxi City.

## Case presentation

### Case 1

**Fig. 1:**
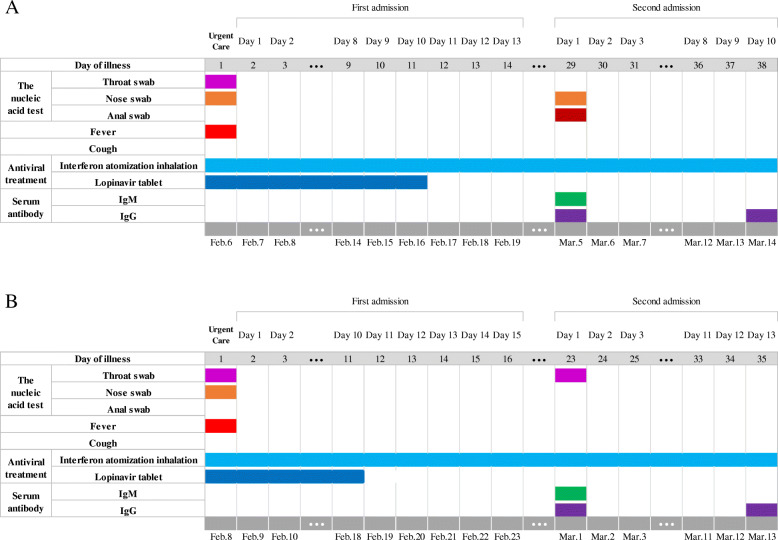
Timeline of the patients with COVID-19 after the onset of illness. **a**, the 8-year-old boy; **b**, the 46-year-old woman

An 8-year-old boy was admitted to the hospital on February 6, 2020, after being quarantined because he had dinner with an infected patient and tested positive for SARS-CoV-2 nucleic acid by a throat swab. The patient had a fever on the first day of admission but no cough, chest tightness or other symptoms. The patient also did not have cardiovascular disease, diabetes or other underlying diseases. Chest CT examination showed nodules in the lower lobe of the right lung, without manifestations of inflammation. Laboratory tests excluded *Mycoplasma pneumoniae* and other causes of viral pneumonia, such as influenza A virus H1N1, H1N1 (2009), H3N2, H5N1, H7N9, influenza B virus (BV and BY types), human coronavirus (229E/HKU1/OC43/NL63/SARS/MERS), parainfluenza virus (1–3), and rhinovirus A/B/C. Routine blood tests showed a white blood cell (WBC) count of 4.53 × 10^9^/L, lymphocyte ratio of 32.0%, and C-reactive protein (CRP) level of 2.2 mg/L. Blood gas tests showed a PaO_2_ of 99 mmHg and a PaO_2_/FiO_2_ of 1.28. Blood chemistry revealed an alanine aminotransferase (ALT) level of 28 U/L, aspartate aminotransferase (AST) level of 30, urea level of 3.5 mmol/L, creatinine level of 35 μmol/L, D-dimer (D2) level of 0.27 μg/L, and creatine kinase myocardial isoenzyme-muscle/brain (CK-MB) level of 14 U/L. The diagnosis was COVID-19 (mild type). After admission, the patient was treated with interferon atomization inhalation (5 million units each time, twice a day) and lopinavir tablets [2 capsules each time (50 mg each capsule), twice a day]. Two days later, his transaminase levels were elevated, and silybin capsule was added to protect the liver. From February 15th to 17th, nucleic acid testing of three consecutive swabs of his nose, pharynx and anus all showed negative results, and his aminotransferase level was reduced (ALT:42, AST:28). The patient was discharged from the hospital and sent to the local Community Health Service Center where patients reside during continued isolation*.* The patient continued atomized recombinant interferon. Two weeks later, nucleic acid re-examination of nose and (rectal) swabs showed positive results; the throat swab was negative. The patient was immediately admitted to the hospital (Fig. 1a). There was still no sign of inflammation on imaging examination (Figure S2). Serum antibody detection showed weak positive IgM antibodies and IgG antibody positivity. Routine blood tests showed a WBC count of 4.05 × 10^9^/L, lymphocyte ratio of 50.6%, and CRP level of 0.5 mg/L (Figure S3A-D). The patient did not receive another treatment except for continuous atomizing with recombinant interferon. Nucleic acid testing of three consecutive swabs of the nose, pharynx and anus was negative after 7 days. All re-examination tests were normal, and the serum antibody test showed IgM antibody negativity and IgG antibody positivity. The patient was allowed to leave the hospital and go to the local Community Health Service Center for continued isolation. After 2 weeks and 4 weeks, all indicators of the patient’s re-examination were normal, and the patient was released from isolation after recovery.

### Case 2

On January 27, a 46-year-old woman had been dining (at the same table) for several consecutive days with a patient who was definitively diagnosed with the new coronavirus infection. During the period of isolation, the nucleic acid test of her throat swab was found to be positive, and she was admitted to the hospital on February 8, 2020 (Fig. 1b). She had a fever on the first day of admission but no cough, chest tightness or other symptoms. She did not have cardiovascular disease, diabetes or other underlying diseases. Chest CT examination showed scattered thin patchy shadows and inflammatory manifestations in both lungs. Laboratory tests excluded *Mycoplasma pneumoniae* and other causes of viral pneumonia, such as influenza A virus H1N1, H1N1 (2009), H3N2, H5N1, H7N9, influenza B virus (BV and BY types), human coronavirus (229E/HKU1/OC43/NL63/SARS/MERS), parainfluenza virus (1–3), and rhinovirus A/B/C. Routine blood tests showed a WBC count of 6.70 × 10^9^/L, lymphocyte ratio of 24.9%, and CRP level of 0.5 mg/L. Blood gas tests showed a PaO_2_ of 99 mmHg and PaO_2_/FiO_2_ of 0.99. Blood chemistry results were as follows: ALT level of 24 U/L, AST28, urea level of 3.0 mmol/L, creatinine level of 46 μmol/L, D2 level of 0.27 μg/L, and CK-MB level of 12 U/L (Figure S3E-H). The diagnosis was COVID-19 (moderate type). After admission, the patient was treated with interferon atomization inhalation (5 million units each time, twice a day) and lopinavir tablets [2 capsules each time (50 mg each capsule), twice a day], in addition to traditional Chinese medicine (Qingfei Paidu Decoction) as an auxiliary treatment. The patient tested negative for nucleic acid by swabs of the nose, pharynx and anus three times on February 17th, 18th, and 19th; imaging of the two lungs revealed a scattered thin film, which was more absorbed than before. The patient was discharged from the hospital on the 23rd and then sent to the local area for continued isolation. One week later, nucleic acid re-examination using nose and (rectal) swabs showed positive results, though the throat swab was negative; the patient was immediately admitted to the hospital. Imaging examination showed the scattered thin film in the two lungs, with little change compared with the previous imaging results (Figure S4). Serum antibody detection indicated weak positivity for IgM antibodies and positivity for IgG antibodies. Routine blood tests showed a WBC count of 3.30 × 10^9^/L, lymphocyte ratio of 32.8%, and CRP level of 8.04 mg/L. The patient did not receive another treatment after readmission except for continuous atomizing with recombinant interferon. Nucleic acid testing of three consecutive swabs of the nose, pharynx and anus were all negative on March 11th, 12th, and 13th. Imaging revealed basic absorption of both lung lesions. The serum antibody test was negative for IgM antibodies and positive for IgG antibodies, and routine blood and blood chemistry tests were normal. The patient was discharged from the hospital and sent to the local Community Health Service Center for continued isolation. After 2 weeks and 4 weeks, all re-examination tests were normal, and the patient was released from isolation after recovery.

## Discussion and conclusions

The cases in this study involved a child with mild-type COVID-19 and an adult female with moderate-type COVID-19 who were discharged from the hospital after three consecutive negative RNA tests but had recurrence of SARS-CoV-2 RNA positivity within 2 weeks and 1 week, respectively. The nucleic acid testing was conducted using three samples, i.e., nose, pharynx and anus swabs, and two brands of detection reagents were used at the same time. In addition, the tests were conducted in the hospital and local Center for Disease Control and Prevention (CDC). All of above measures might eliminate interference caused by specimen collection and transport, detection reagent, personnel technology and other factors [8, 9]. When the patients were positive for recurrence, we detected serum antibody levels over time. By analysing the patient’s symptoms, antibodies, and dynamic changes in imaging and haematology, we found that the patients showed a trend of gradual improvement and recovery throughout treatment.

With regard to the recurrence of positivity, we consider the following. Although the patients met the existing discharge standard of care, the virus may have still been present in the lower respiratory tract. The use of antiviral drugs effectively inhibited replication of the virus in the patients, and with the decrease in the number of viruses, the available nucleic acid detection reagent was not able to effectively detect the low virus titre of upper respiratory tract samples. After the patient was discharged from the hospital, the withdrawal or reduction of antiviral drugs caused the virus to remain in the patient’s body for a short time, after which a nasal swab was once again positive in a short period of time. With the appearance of antibodies and the recovery of immune function, most patients can be cured without treatment. We recommend follow up without readmission if such patients have no symptoms. It is worth suggesting that at the first discharge from the hospital, patients might still be infectious to a certain degree. At present, it is of great significance to prevent the recurrence of positive SARS-CoV-2 nucleic acid tests in patients; however, panic is not necessary. Hence, improving the discharge standard of care is highly recommended. Three consecutive negative results were obtained at the medical institutions, and the CDC in the region found at least one positive result*.* At the same time, dynamic monitoring of serum antibodies and imaging should be promoted. It is suggested that the ability of patients to infect others should be evaluated by serum antibody detection, virus culture and isolation of throat swab. The designated hospital should make good contact with the primary medical institutions where the patient resides, share the medical records, and provide information of discharged patients to the primary medical and health institutions in the patient’s jurisdiction or residence in a timely manner. This will also help to reduce the cost of patient care. To strengthen the management of discharged patients, it is suggested to continue isolation management and health monitoring for 14 days after discharge, wear masks, live in an unshared room with good ventilation, reduce close contact with family members, prepare and eat meals in isoaltion perform hand hygiene, and avoid outside the house. At the same time, it is suggested that patients should be followed up in the second and fourth weeks after discharge.

## Supplementary Information

**Additional file 1:** **Table S1.** Clinical classifications. **Figure S1.** Epidemiologic links of severe acute respiratory syndrome coronavirus 2 infection within a cluster. **Figure S2.** Chest CT images of the 8-year-old boy with COVID-19. A, The first day of admission: nodules in the right lungs without manifestations of inflammation. B, The day of first discharge: nodules in the right lungs and without manifestations of inflammation. C, The day of readmission: nodules in the right lungs and without manifestations of inflammation. D, The day of discharge from the hospital: nodules in the right lungs and without manifestations of inflammation. **Figure S3.** Levels of WBC, lymphocyte ratio, CRP and ALT in the two cases fluctuated with the illness day. (A-D) The 8-year-old boy; (E-H) the 46-year-old woman. WBC, white blood cell count; CRP, C-reactive protein; ALT, alanine aminotransferase. **Figure S4.** Chest CT images of the 46-year-old woman with COVID-19. A-B, The first day of admission: scattered thin patchy shadow and inflammatory manifestations in both lungs. C-D, The day of first discharge: the two lungs showed a scattered thin film, which was more absorbed than before. E-F, The day of readmission: two lungs showed a scattered thin film, with little change compared with the previous imaging result. J-H, The day of discharge from hospital: basic absorption of both lung lesions

## Data Availability

Data sharing is not applicable to this article as no datasets were generated or analysed during the current study.

## Abbreviations

SARS-CoV-2: Severe acute respiratory syndrome coronavirus 2
COVID-19: Coronavirus disease 2019
WHO: World Health Organization
CDC: Center for Disease Control and Prevention
WBC: White blood cell
CRP: C-reactive protein
CK-MB: Creatine kinase myocardial isoenzyme-muscle/brain
ALT: Alanine aminotransferase
AST: Aspartate aminotransferase
D2: D-dimer
