# Supplementary material for: Recurrence of SARS-CoV-2 nucleic acid positive test in patients with COVID-19: a report of two cases
Source: BMC Pulm Med. 2020 Nov 23;20:308. doi: 10.1186/s12890-020-01348-8 (PMC7681189; doi:10.1186/s12890-020-01348-8)
Supplement: Supplementary file 1 — Additional file 1: Table S1. Clinical classifications. Figure S1. Epidemiologic links of severe acute respiratory syndrome coronavirus 2 infection within a cluster. Figure S2. Chest CT images of the 8-year-old boy with COVID-19. A, The first day of admission: nodules in the right lungs without manifestations of inflammation. B, The day of first discharge: nodules in the right lungs and without manifestations of inflammation. C, The day of readmission: nodules in the right lungs and without manifestations of inflammation. D, The day of discharge from the hospital: nodules in the right lungs and without manifestations of inflammation. Figure S3. Levels of WBC, lymphocyte ratio, CRP and ALT in the two cases fluctuated with the illness day. (A-D) The 8-year-old boy; (E-H) the 46-year-old woman. WBC, white blood cell count; CRP, C-reactive protein; ALT, alanine aminotransferase. Figure S4. Chest CT images of the 46-year-old woman with COVID-19. A-B, The first day of admission: scattered thin patchy shadow and inflammatory manifestations in both lungs. C-D, The day of first discharge: the two lungs showed a scattered thin film, which was more absorbed than before. E-F, The day of readmission: two lungs showed a scattered thin film, with little change compared with the previous imaging result. J-H, The day of discharge from hospital: basic absorption of both lung lesions [file 12890_2020_1348_MOESM1_ESM.docx]

**Supplementary Appendix**

**Recurrence of SARS-CoV-2 nucleic acid positive in patients with COVID-19: Two Cases Report**

Jian Wu^1,2^**^†^**, Juan Cheng^3†^, Xiaowei Shi^1^**^†^**, Jun Liu^4^**^†^**, Biao Huang^5^, Xinguo Zhao^6^, Yuanwang Qiu^7^, Jiong Yu^1^, Hongcui Cao^1,8^*, Lanjuan Li^1^

^1^State Key Laboratory for the Diagnosis and Treatment of Infectious Diseases, National Clinical Research Center for Infectious Diseases, The First Affiliated Hospital, College of Medicine, Zhejiang University, 79 Qingchun Rd., Hangzhou 310003, China;

^2^Department of Laboratory Medicine, Yancheng Clinical Medical College of Nanjing Medical University, Yancheng 224001, China;

^3^Department of Infectious Disease, The Second People’s Hospital of Yancheng City, Yancheng 224005, China;

^4^Department of Laboratory Medicine, The Fifth People’s Hospital of Wuxi, Wuxi214005, China;

^5^College of Life Sciences and Medicine, Zhejiang Sci-Tech University, Hangzhou, 310018, China;

^6^Department of Respiration, The Fifth People’s Hospital of Wuxi, Wuxi214005, China;

^7^Department of Infectious Diseases, The Fifth People’s Hospital of Wuxi, Wuxi214005, China;

^8^Zhejiang Provincial Key Laboratory for Diagnosis and Treatment of Aging and Physic-chemical Injury Diseases, 79 Qingchun Rd, Hangzhou 310003, China.

**^†^** These authors contributed equally to this work

***To whom correspondence should be addressed:**

Hongcui Cao, M.D. State Key Laboratory for the Diagnosis and Treatment of Infectious Diseases, National Clinical Research Center for Infectious Diseases, The First Affiliated Hospital, College of Medicine, Zhejiang University, 79 Qingchun Rd., Hangzhou 310003, China; Tel: 86-571-87236451; Fax: 86-571-87236459;

Email: [hccao@zju.edu.cn](mailto:hccao@zju.edu.cn)

**Definition of cases**

We defined the COVID-19 patients according to the epidemiological history and consistent with any two clinical manifestations and the pathogenic evidence, as previously described.^1^ Real time-PCR was used to detect the coronavirus RNA. ^1^ The definition of clinical classifications was described in detail in the Table S1.

**Data collection**

We collected all the data of two cases including clinical, demographic, epidemiolocal history, laboratory parameters, chest CT, length of hospitalization, prognosis et al. At the time of admission, all the cases were routine tested in the laboratory, including blood routine, blood biochemistry, coagulation function and so on. The epidemiolocal history of two cases was followed up as in Figure S1.

**Table S1. Clinical classifications**

| **Type** | **Criteria** |
| --- | --- |
| Mild | the clinical symptoms were light, and there was no sign of pneumonia on imaging |
| Moderate | with fever, respiratory tract and other symptoms, imaging suggests pneumonia |
| Severe | coincide with any of the following: (1) respiratory distress, respiration rate (RR) ≥ 30 times / min; (2) the oxygen saturation ≤ 93% in the resting state; (3) PaO_2_ / FiO_2_ ≤ 300 mmHg (1mmHg = 0.133 kPa) |
| Critically ill | coincide with any of the following: (1) respiratory failure occurs and mechanical ventilation is required; (2) shock; (3) the patient develops other organ failure and needs ICU monitoring and treatment |

**Figure S1** **Epidemiologic linkage of severe acute respiratory syndrome coronavirus 2 infection within a cluster.**


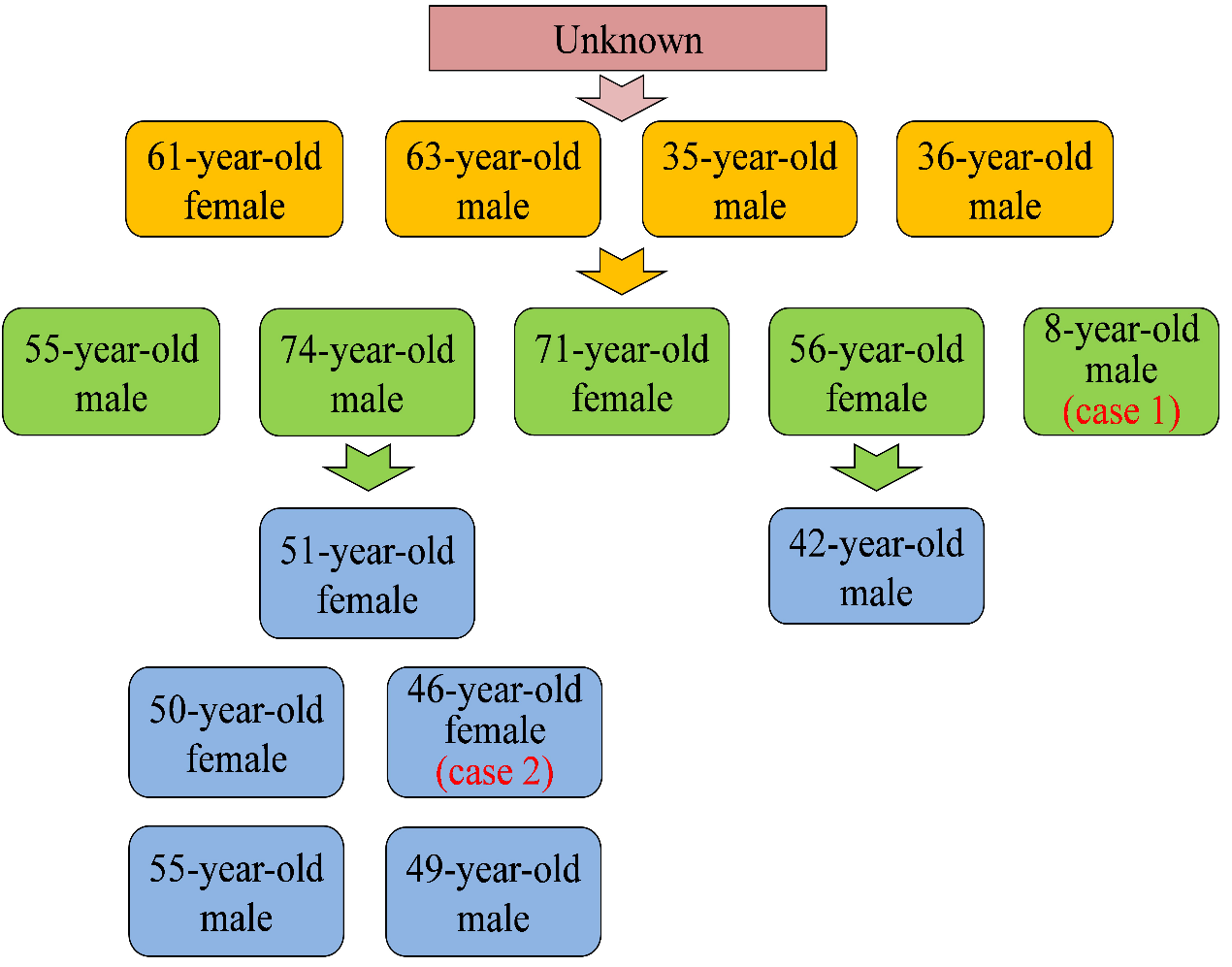


**Figure S2 Chest CT images of the 8-year-old boy with COVID-19.**

A, The first day of admission: nodules in the right lungs and without manifestations of inflammation; B, The day of first discharge: nodules in the right lungs and without manifestations of inflammation; C, The day of readmission: nodules in the right lungs and without manifestations of inflammation; D, The day of discharged from hospital: nodules in the right lungs and without manifestations of inflammation.


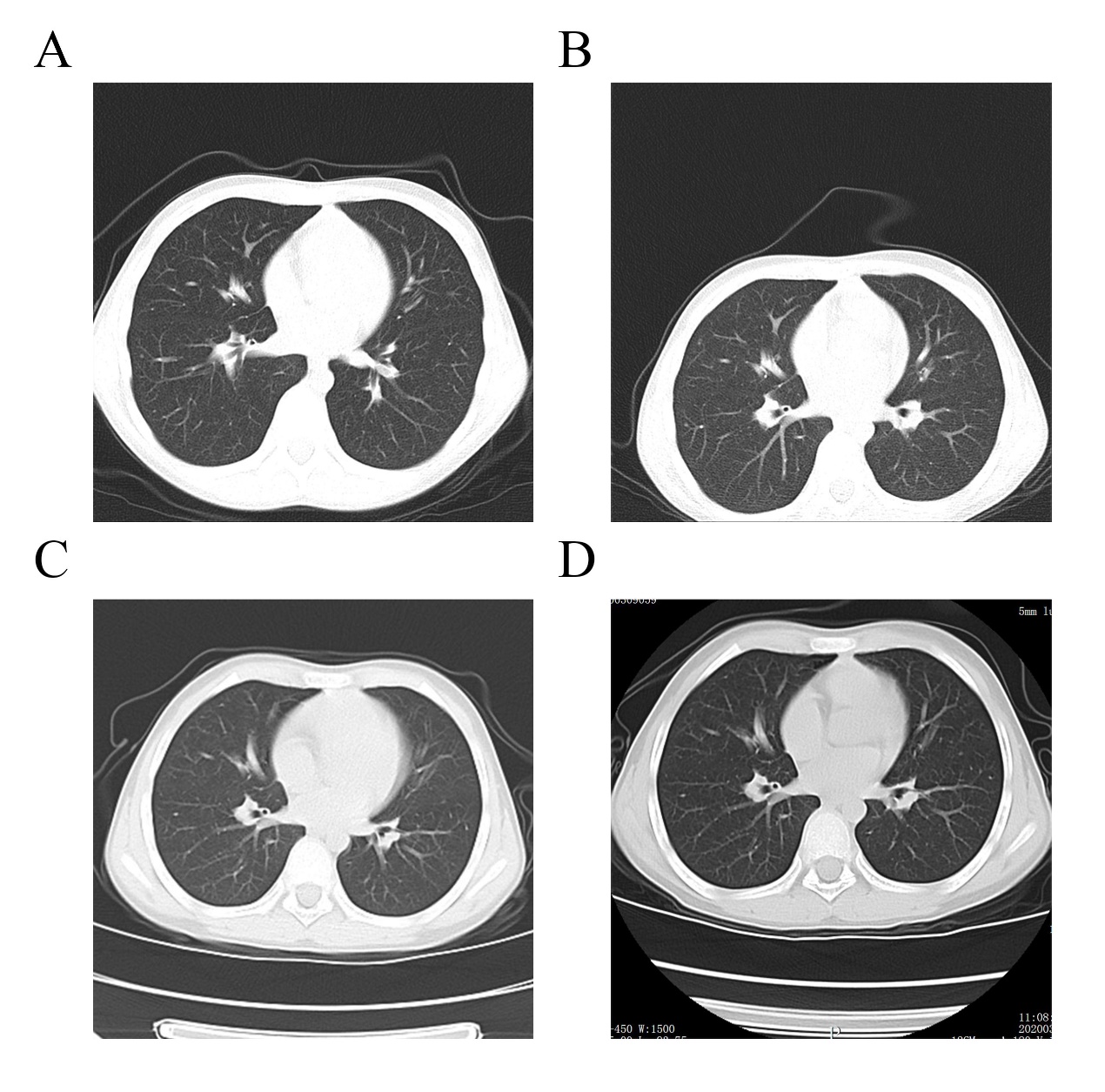


**Figure S3 The levels of WBC, lymphocytes ratio, CRP and ALT of two cases fluctuates with the illness day.** (A-D) the 8-year-old boy; (E-H) the 46-year-old woman. WBC, white blood cell count; CRP, C-reactive protein; ALT, Alanine aminotransferase.


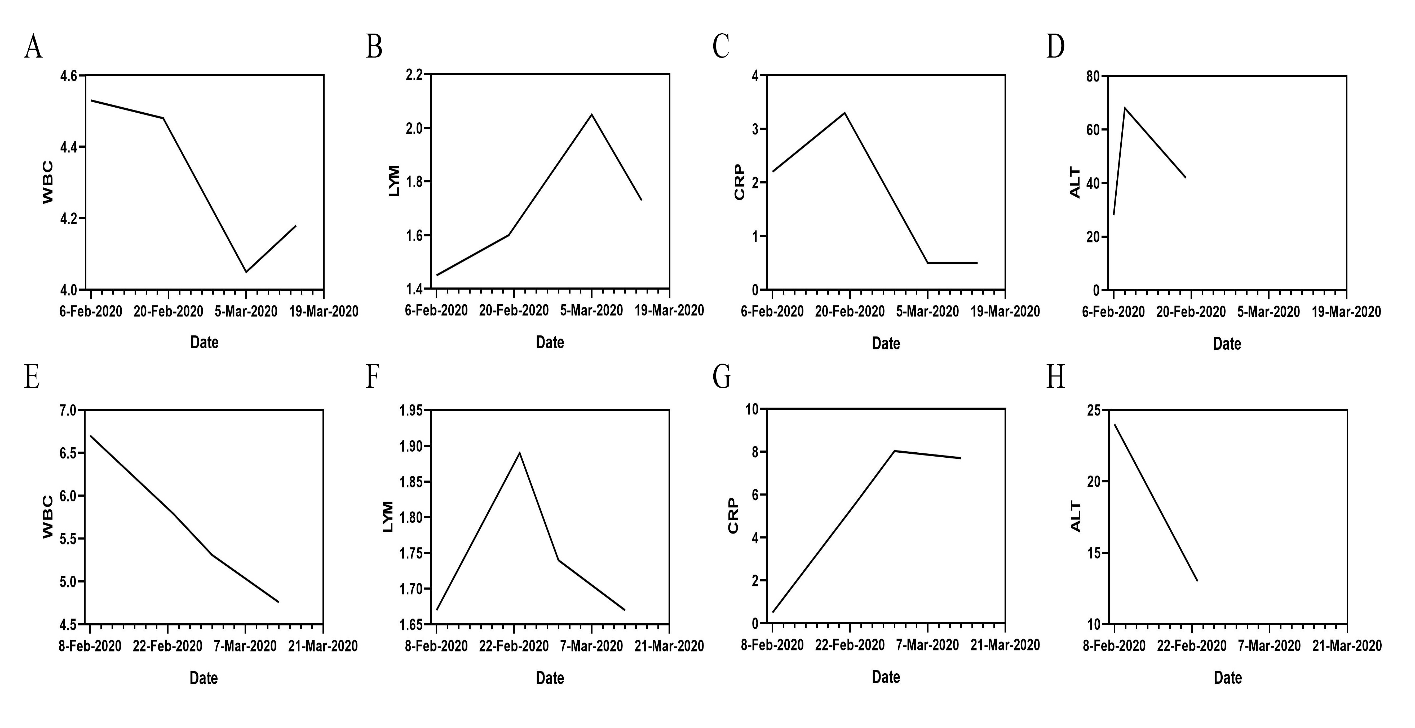


**Figure S4 Chest CT images of the 46-year-old woman with COVID-19.**

A-B, The first day of admission: scattered thin patchy shadow and inflammatory manifestations in both lungs; C-D, The day of first discharge: the two lungs were scattered in a thin film, which was more absorbed than before; E-F, The day of readmission: two lungs were scattered in a thin film, with little change compared with the previous one; J-H, The day of discharged from hospital: basic absorption in both lung lesions.


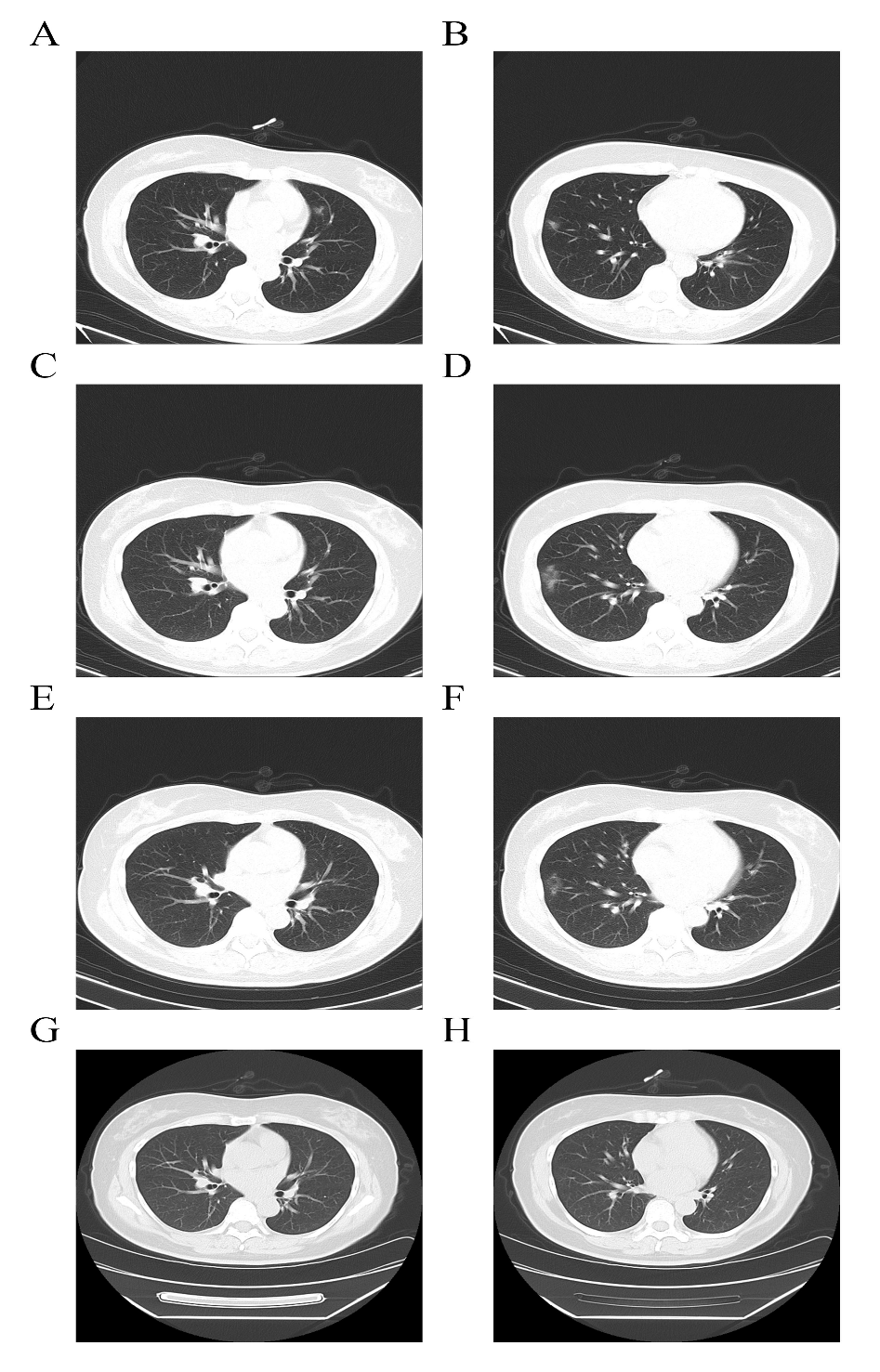


**References**

1. Wu J, Liu J, Zhao X, et al. Clinical Characteristics of Imported Cases of COVID-19 in Jiangsu Province: A Multicenter Descriptive Study. Clinical Infectious Diseases. 2020. doi: 10.1093/cid/ciaa199
